# Round the Hospitals

**Published:** 1916-05-13

**Authors:** 


### ROUND THE HOSPITALS.

May 12 was the anniversary of Florence Nightingale's birthday. Her statue in Waterloo Place has been taken down for alterations, or it would no doubt have been covered with wreaths as it was last year. The Dean of St. Paul's has, however, very kindly consented that any member of the nursing profession in uniform could have free admission to the Crypt on Friday, May 12, 1916. Trained nurses may thus make a pilgrimage to the most beautiful memorial by Mr. Arthur G. Walker, which probably has been ever erected to the memory of a great woman. The College of Nursing should provide means to keep Florence Nightingale's memory ever green.

The College of Nursing will have many nuts to crack, and amongst them not the least easy will be the questions arising as to the position and future of the smaller training-schools. Dr. Hurd states that registration has tended to raise the status of graduates from this type of school in the United States. Sister Félicie Norton, in our correspondence columns, advocates affiliation between various types of hospitals. She raises a question which has been under discussion for some time, and which may possibly lead to developments tending to simplify the solution of the problems presented. We have opened our columns to correspondence on the position of the small hospitals and schools, believing that if those who are interested and possessed of knowledge, both of the small schools and of the position and experience of those who may have entered them for training, will state their views, the result will be much valuable information that cannot fail to prove helpful for many reasons.

We have great pleasure in congratulating Miss Mary S. Rundle on her appointment as secretary to the College of Nursing. There were many excellent candidates, a fact which affords satisfactory evidence of the large number of highly educated and trained women who have qualified as workers in the field of nursing. Miss Rundle's training has been extensive. She has had the enormous advantage of close association with, and the guidance and instruction of the greatest American authority on nursing matters, Miss Nutting, of Columbia University. The appointments Miss Rundle has held have made her familiar with many aspects of administration and of the training of nurses, which cannot fail to prove of great assistance to her in the discharge of the onerous and responsible duties which will devolve upon her in connection with the College. Miss Rundle will be warmly welcomed by workers throughout the hospital world, who, she may confidently rely, will gladly give her all possible help in their power. We congratulate the Council upon this appointment, which we anticipate will be abundantly justified by results.

A contemporary has caused some misapprehension and trouble by stating that a meeting would be held on the 12th instant, when it was expected that the constitution of the Consultative Board would be advanced a stage further. This statement had no foundation in fact, and we are advised that no such meeting can be held until at least a month after the date, April 18, attached to Mr. Stanley's manifesto to the hospitals and nurse-training schools. It is satisfactory to learn that practical unanimity in support of the College has been secured in Scotland, largely from the efforts of Professor Glaister and Miss Gill, and that a further important meeting of hospital and training-school authorities in Scotland has been arranged for an early date. The practically unanimous opinion in favour of the College which is manifesting itself throughout England and Wales is as important as it is gratifying.

About fifty Guy's nurses assembled in the Nurses' Home of Guy's Hospital on Friday, May 5, for the annual dinner of the Guy's Hospital Nurses' League. The decrease in the numbers of those present, which was, of course, due to the war, was made up for by the great friendliness of spirit that prevailed. The question so often asked by nurses who had not met for years, "What are you doing now?" was heard on all sides; and the reply "Oh, I'm only married," made by one thus questioned expressed something of the appreciation felt generally for those Guy's nurses both at home and abroad who are working hard, often under great difficulties, and are earning and sometimes winning high honours. The keynote of the meeting held after dinner was appreciation for Miss Swift's work as Matron of the British Red Cross Society, of congratulation for the honours bestowed upon her, and pride at the very important part she has played in the inception of the College of Nursing. It was a great joy to be able to listen to Miss Swift speaking to her nurses, with an intimacy that no other gathering could have induced, of all that the College might do for nurses. Miss Swift mentioned that both the Principal Matrons in France and in Egypt were instructing the nurses on active service concerning the aims and constitution of the College, and ascertaining their wishes and views on the subject. Miss Swift said that matrons at home would be betraying their co-workers abroad if they refrained from doing their share in working for the College. Miss Haughton gave an account of the College of Nursing, and brought out the fact that the matrons of the larger training schools are uniting as never before to help the smaller ones to greater efficiency. As one heard Miss Haughton speaking a vision arose of those matrons who are working for the College standing on a daïs and saying to other matrons, "Come and stand here beside us. We should like you to be able to give your nurses all the advantages that we have won for ours"; and the audience felt proud of their leaders and of Guy's.

Our readers will be interested in the eleventh article on "Textile Supplies for Hospitals," which appears on p. 157. There is a final article to conclude the series, and we should be glad if matrons or sisters, or both, would let the Editor know how far these articles have proved helpful, and whether a reprint of them for the desk will be calculated to have for them a permanent value. The article on the April examination of the Incorporated Society of Trained Masseuses, on p. 156, should have a practical interest for matrons everywhere.

We have already noted that the matron of the Norfolk and Norwich Hospital has, by making use of voluntary labour in the laundry, been able to deal with all the extra work entailed by the military wards without extra expense. This seems to be an excellent way of making use of the services of ladies who, owing to home ties, are not able to do munition service and prefer hard physical work to other less energetic ways of doing war service. Laundry labour is suitable for helpers who cannot give all their time to the work, and a little method in arranging the hours of duties will make it possible for the regular staff to be constantly assisted by two or three extra pairs of hands working in short shifts. We congratulate Miss Cann on having conceived this means of voluntary help.

A disabled soldier was being helped to alight from a train by a hale and hearty old porter. As the latter looked at the brave young face and the poor maimed body, tears of sympathy dimmed his eyes. The soldier laid the one hand which remained to him on the porter's shoulder and said with a smile, "It's all right, old chap; if I don't worry, why should you?"

For Matrons' and Sisters' Appointments see page vi.

